# Target sequencing of cancer-related genes in early esophageal squamous neoplasia resected by endoscopic resection in Japanese patients

**DOI:** 10.18632/oncotarget.26397

**Published:** 2018-12-04

**Authors:** Shoji Kobayashi, Tatsuya Yamaguchi, Shinya Maekawa, Shinichi Takano, Toru Kuno, Keisuke Tanaka, Yuya Tsukui, Fumihiko Iwamoto, Takashi Yoshida, Yukiko Asakawa, Mitsuharu Fukasawa, Yasuhiro Nakayama, Taisuke Inoue, Tomoyoshi Uetake, Minoru Sakamoto, Masahiko Ohtaka, Tadashi Sato, Nobuyuki Enomoto

**Affiliations:** ^1^ First Department of Internal Medicine, Faculty of Medicine, University of Yamanashi, Chuo, Yamanashi 409-3898, Japan

**Keywords:** next generation sequencing, early esophageal squamous neoplasia, cancer-related gene mutation, copy number variation

## Abstract

**Background and Aims:**

Next generation sequencing (NGS) has revealed a great deal about cancer-related somatic changes in esophageal squamous cell neoplasia; however, the changes in the very early stages remain unclear.

**Results:**

*TP53* (87%) and *CDKN2A* (20%) hot spot mutations were frequently found in early lesions. *TP53* was the most common mutation (LGIN/HGIN, 86%; EP, 83%; LPM, 95%; MM/SM1, 80%), followed by *CDKN2A* (29%, 28%, 16% and 10%, respectively); the frequency of other mutations increased as the disease advanced (*p* < 0.01). Copy number variation analysis revealed copy number aberrations in multiple genes, including *PIK3CA* amplification (48%). NGS was superior to p53 immunostaining for detecting *TP53* mutations (74% vs. 87%); in combination, the two tests improved detectability to 94%. Clinically, smoking was associated with the occurrence of *TP53* mutations in these early lesions (*p* = 0.049).

**Materials and Methods:**

Fifty-four early esophageal neoplasia lesions from 47 patients treated by endoscopic resection (low-grade intraepithelial neoplasia [LGIN], *n =* 1; high-grade intraepithelial neoplasia [HGIN] *n =* 7; invasion limited to epithelium [EP/M1], *n =* 18; lamina propria mucosae [LPM/M2], *n =* 19; muscularis mucosae [MM/M3], *n =* 8; and upper third of the SM [SM1], *n =* 2) were isolated from formalin-fixed paraffin-embedded tissue specimens by laser-capture microdissection. Target sequencing of 50 cancer-related genes was performed with an Ion Proton sequencer; their association with the clinical characteristics was investigated.

**Conclusions:**

Mutations of *TP53* and *CDKN2A*, and *PIK3CA* amplification were common in early esophageal squamous neoplasia, while other mutations accumulated with disease progression. An understanding of these molecular events might provide a molecular basis for early lesion treatment.

## INTRODUCTION

Esophageal carcinoma is one of the most progressive types of cancer, and is the sixth and seventh leading cause of cancer-related mortality in the world and in Japan, respectively. While adenocarcinoma is the predominant form of esophageal cancer in Western countries, esophageal squamous cell carcinoma (ESCC) is the most prevalent histological form in Asian countries, including Japan [1, 2].

Due to recent advances in next generation sequencing (NGS) technology, which has facilitated analysis of the whole genome, exome and target sequences, a great deal of information on the aberrations of cancer-related genes causing the development and progression of ESCC has accumulated, and this has led to the description of the landscape of ESCC-related somatic aberrations, such as *TP53, CDKN2A, NOTCH* and *PIK3CA* [3–8]. Most previous NGS-based studies have focused on advanced ESCCs. At present, the changes in early ESCC, and the genetic events occurring at the earliest stage of ESCC are not well understood.

In this background, the two most recent NGS-based studies from China reported the frequent presence of hotspot mutations as well as copy number aberrations in the early stages of ESCCs [3, 9]. These observations were rather astonishing, since intraepithelial neoplasias (IENs) are generally considered to be premalignant, and treatment is not actively recommended [10]. If such somatic aberrations were present, early treatment would be more reasonable. However, in these reports, most IENs were surgically resected *en bloc* with the adjacent advanced ESCC tissues. IENs without any adjacent advanced lesions would be more appropriate for analysis, since it is unclear whether IENs that are adjacent to advanced lesions share similar aberration profiles with those that are not.

The recent development of therapeutic endoscopic procedures has enabled the *en bloc* resection of esophageal squamous neoplasia by endoscopic submucosal dissection (ESD) and/or endoscopic mucosal resection (EMR) [11, 12]. In Japan, early esophageal lesions (intraepithelial neoplasia [IEN], invasion limited to the epithelium [EP], invasion limited to the lamina propria mucosae [LPM/M2], and invasion limited to the muscularis mucosae [MM/M3]) are increasingly considered to be appropriate targets for ESD. However, among IENs, low-grade intraepithelial neoplasia (LGIN) and high-grade intraepithelial neoplasia (HGIN) are often classified as premalignant in Western countries and their malignant potential has not yet been well characterized [10]. ESD and EMR target early neoplastic lesions and tumor tissues can be obtained *en bloc*; thus, early esophageal specimens resected by ESD or EMR provide ideal materials for cancer-related gene analysis of very early esophageal neoplasia.

In the present study, to investigate the correlation between the somatic changes in cancer-related genes and the very early stages of esophageal squamous neoplasia, we analyzed formalin-fixed paraffin-embedded (FFPE) specimens that had been resected by ESD or EMR, by NGS-based target sequencing using the Ion Proton-based cancer panel platform. In addition, in order to disclose the somatic aberrations, we introduced laser capture microdissection (LCM) technology, which allowed us to obtain minute neoplastic samples precisely.

## RESULTS

### Target deep sequencing for somatic mutations of cancer-related genes

We obtained DNA from laser-capture microdissected neoplasia and from the corresponding adjacent non-neoplastic mucosa at an average concentration of 8.5 ng/uL and 5.7 ng/uL, respectively (data not shown). The average ratio of 260/280 nm optical absorbance in these two tissues was 1.8 and 2.1, respectively (data not shown). A total of 93 neoplasia-specific hotspot mutations were found in 54 neoplastic lesions with a cutoff value of 5%. *TP53* mutations were most common (*n* = 47, 51%), followed by *CDKN2A* (*n* = 12, 13%), *APC* (*n* = 4, 4%)*, VHL* (*n* = 3, 3%)*, KDR* (*n* = 3, 3%)*, PTEN* (*n* = 3, 3%)*, CSF1R* (*n* = 3, 3%)*, STK11* (*n* = 2, 2%)*, CTNNB1* (*n* = 2, 2%)*, RB1* (*n* = 2, 2%) and *KIT* (*n* = 2, 2%) mutations.

**Figure 1:**
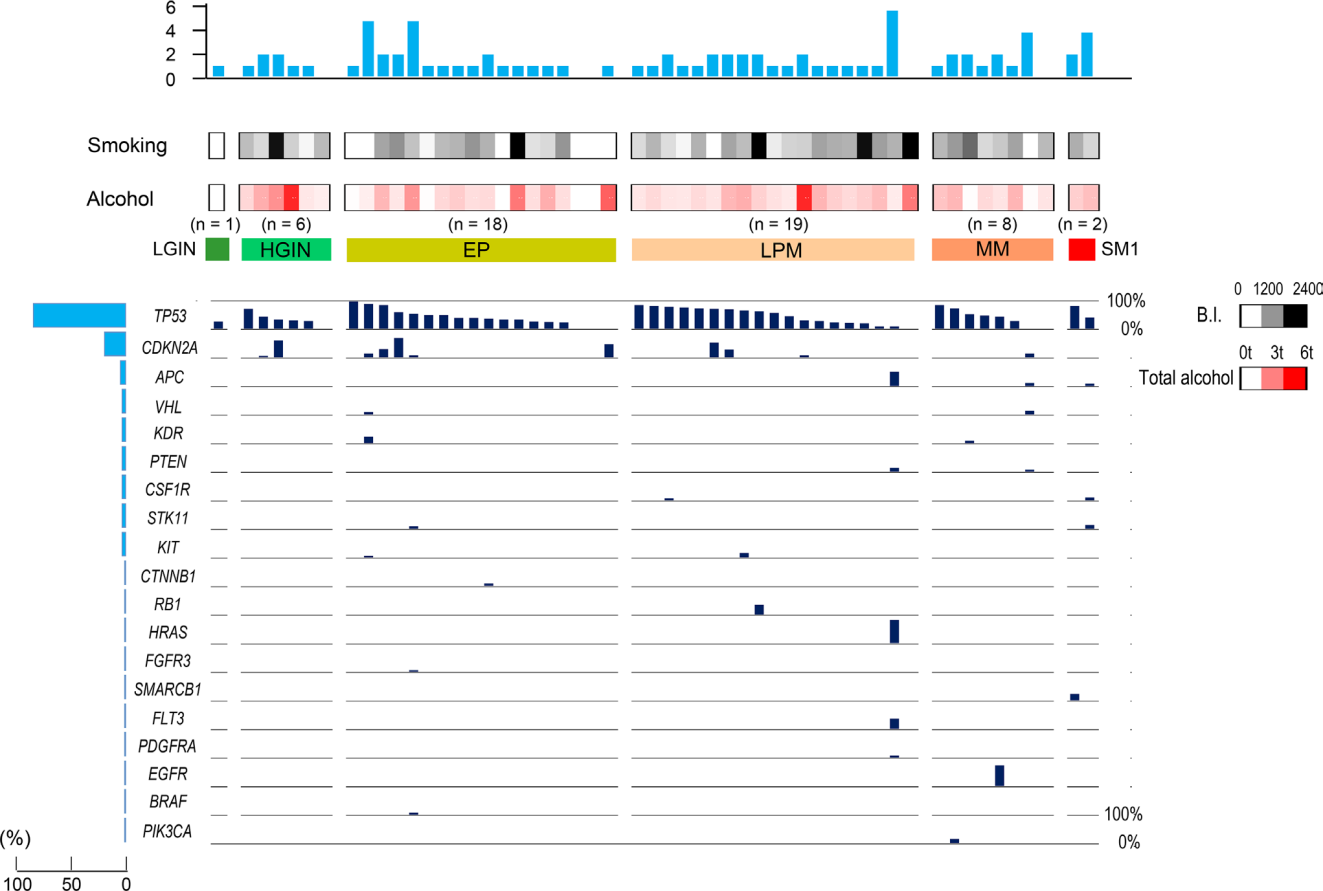
Hot spot cancer-related gene mutations in each stage of esophageal neoplasia The bar length in the main panel indicates the variant frequency. The histograms on the left panel show the percentages of neoplasia lesions with mutations of each gene, and the histograms on the upper panel show the number of mutations. LGIN, low-grade intestinal metaplasia; HGIN, high-grade intestinal metaplasia; EP, invasion limited to the epithelium; LPM, invasion limited to the lamina propria mucosae; MM, tumor invading into the muscularis mucosae; SM1, invasion limited to the upper third of the submucosa.

When the mutations were demonstrated from the viewpoint of individual neoplasia, *TP53* mutations, which were detected in 47 (87%) of 54 lesions were most common. *TP53* mutations were frequently found, irrespective of the tumor depth, even in lesions as early as LGIN or HGIN (Figure 1). With regard to the frequent hotspot mutations, *CDKN2A* was the second most frequent mutation after *TP53*, followed by *APC, VHL, KDR, PTEN, CSF1R, STK11, CTNNB1, RB1* and *KIT* mutations. When the associations among the number of mutated genes other than *TP53*/*CDKN2A*, major cell-cycle regulator genes, and the tumor depth were investigated, these 2nd mutated genes more frequently appeared with deeper lesions, indicating that they played an additional role (*p* < 0.01, Figure 1 and Table 1). In addition to neoplasia, we also collected and evaluated the adjacent background mucosa in all 54 lesions and detected 25 variants in 21 lesions. However, with the exception of one patient (ESCC_42), the mutation frequencies of the background mucosa specimens were less than 5% (Supplementary Table 1).

**Table 1:** Correlation between hotspot mutations and the invasion depth

	LGIN/HGIN	EP	LPM	MM/SM1	*P*-value
*TP53* mutation	6/7 (85%)	15/18 (83%)	18/19 (95%)	8/10 (80%)	0.89
*CDKN2A* mutation	2/7 (29%)	5/18 (28%)	3/19 (16%)	1/10 (10%)	0.19
*TP53 or CDKN2A* mutation	6/7 (85%)	16/18 (89%)	18/19 (95%)	9/10 (90%)	0.85
Other mutations	0/7 (0%)	3/18 (17%)	4/19 (21%)	6/10 (60%)	0.01^*^

Correlation between invasion depth and the mutation rate was calculated by the Cochran-Armitage trend test.

Moreover, we further compared the somatic aberration status between our early esophageal neoplastic lesions and advanced ESCCs (internet-based data). The somatic aberration data of 227 advanced ESCCs in 50 cancer-related genes analyzed in our study was retrieved from the online database (cBioPortal for Cancer Genomics (http://cbioportal.org): mutation data of the Cancer Genome Atlas [TCGA] and International Cancer Genome Consortium [ICGC] were available) [13]. The only evident difference between these two groups was in the expression of three genes: *CDKN2A*, *TP53*, and *VHL* (Supplementary Figure 1).

### Nucleotide substitution patterns

**Figure 2:**
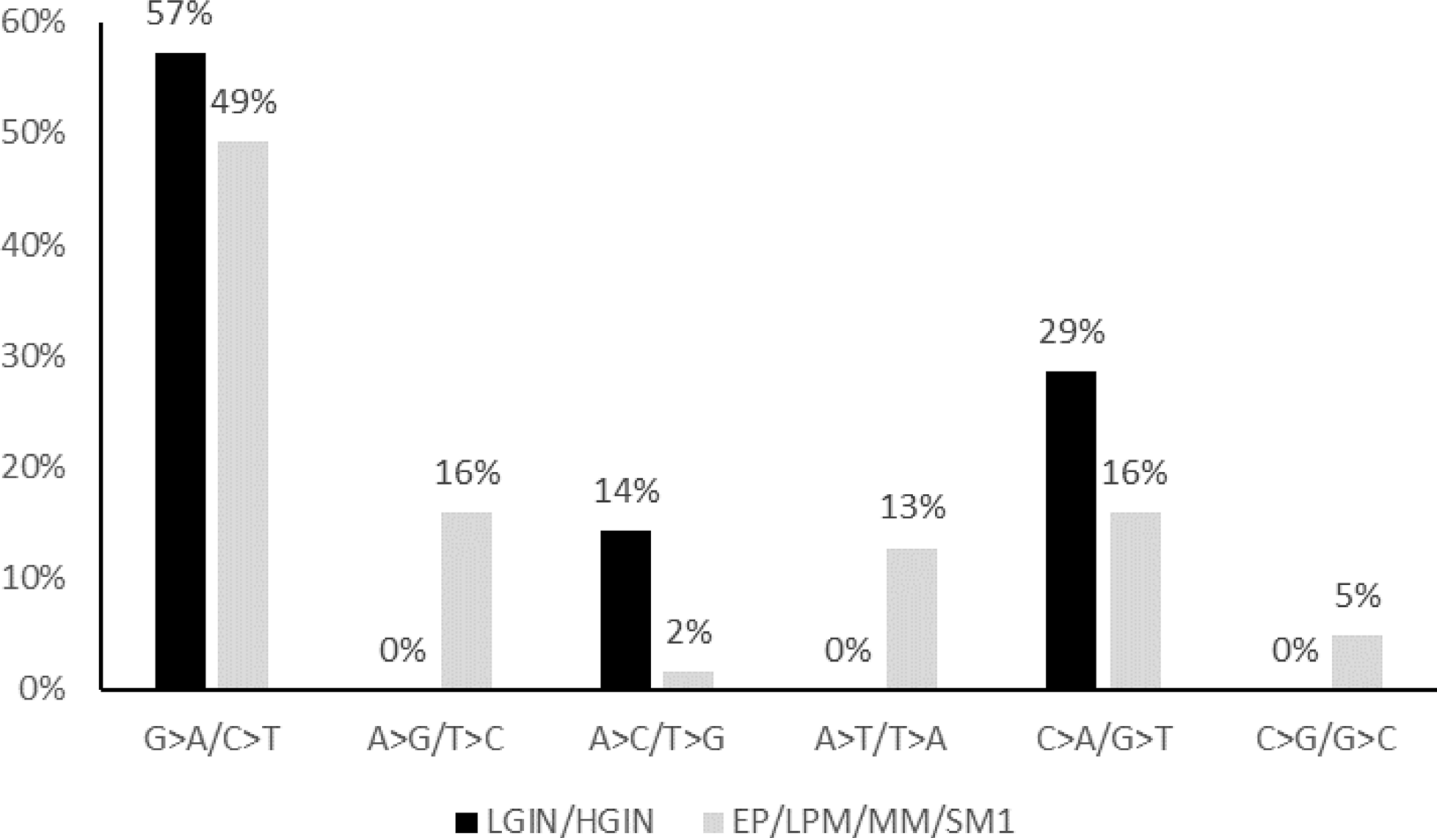
The nucleotide substitution patterns observed in hot spot mutations among LGIN/HGIN and EP/LPM/MM/SM1 LGIN, low-grade intestinal metaplasia; HGIN, high-grade intestinal metaplasia; EP, invasion limited to the epithelium; LPM, invasion limited to the lamina propria mucosae; MM, tumor invading the muscularis mucosae; SM1, invasion limited to the upper third of the submucosa.

The nucleotide substitution patterns in early esophageal squamous neoplasia were investigated. As shown in Figure 2, G>A/C>T transversion was the most frequent nucleotide substitution. The nucleotide substitution patterns of LGIN/HGIN lesions and EP/LPM/MM/SM1 lesions did not differ to a statistically significant extent.

### Copy number variations

**Figure 3:**
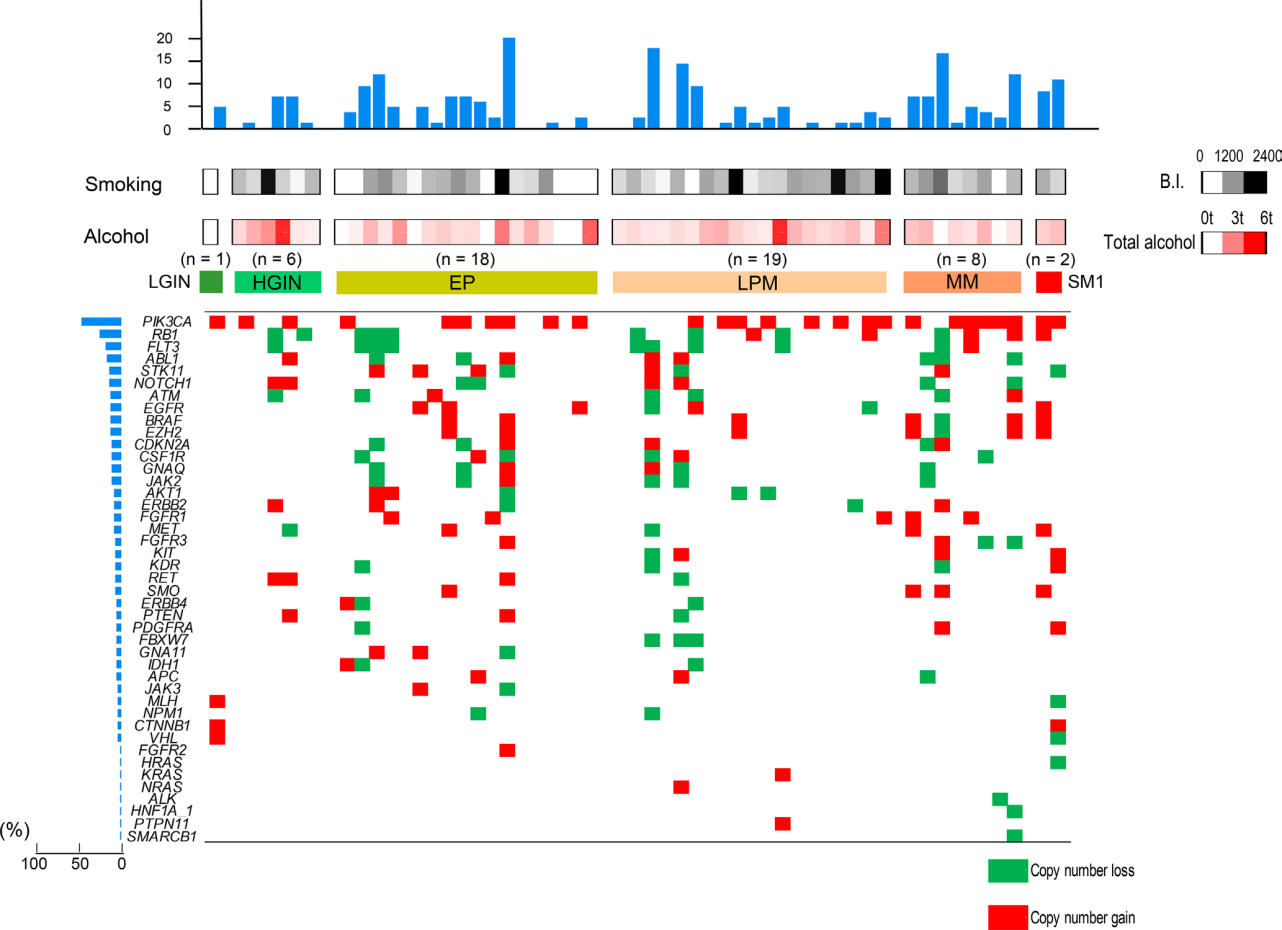
Copy number variations, including copy number gain and loss in each stage of esophageal neoplasia The histograms on the left panel show the percentages of cases with copy number alterations in each gene, and the histograms on the upper panel show the number of copy number alterations in each case. LGIN, low-grade intestinal metaplasia; HGIN, high-grade intestinal metaplasia; EP, invasion limited to the epithelium; LPM, invasion limited to the lamina propria mucosae; MM, tumor invading into the muscularis mucosae; SM1, invasion limited to the upper third of the submucosa.

The copy number variations calculated by the Ion Reporter CNV detection program are shown in Figure 3. Copy number gains or losses were frequently observed in various genes, even in early-stage esophageal lesions, including LGIN/HGIN; the amplification of CNV of *PIK3CA* was most frequently observed (48%, 26/54). When the number of CNVs was compared between IENs and EP/LPM/MM, EP/LPM/MM tended to have more CNVs; however, the difference was not statistically significant (Supplementary Table 2).

### Comparison between TP53 mutations and p53-antibody immunohistochemistry

**Figure 4:**
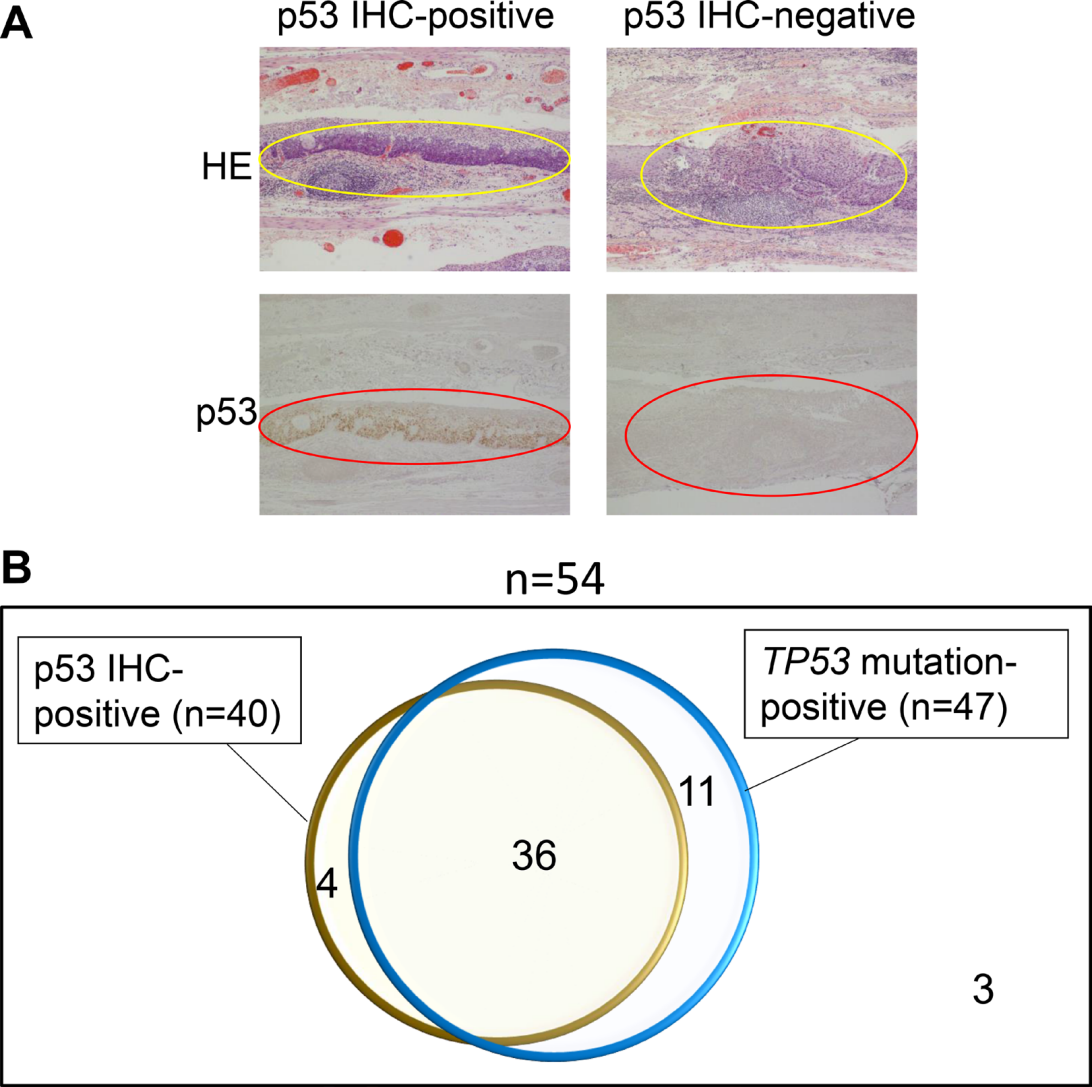
Immunohistochemical (IHC) staining for p53 was performed for 55 esophageal neoplasia specimens and the results were compared to the results of NGS *TP53* mutation analysis Representative examples of p53 IHC-positive neoplasia and p53 IHC-negative neoplasia are shown (**A**). The Venn diagram shows the association between IHC and NGS mutation studies (**B**).

Immunostaining with p53 antibodies was performed in all cases, and the dyeability was evaluated and compared with *TP53* mutations (Figure 4). Forty of 54 (74%) lesions were positively stained. *TP53* mutations were identified in 36 of these 40 p53-antibody-positive lesions (90%). Four immunostaining-positive lesions were not identified by NGS mutational analysis. On the other hand, 47 of 54 lesions (87%) were judged as having *TP53* mutations by NGS, and 11 of 47 lesions with *TP53* mutations were p53-negative. With regard to the reasons for immunostaining-negativity in these 11 lesions, the TP53 mutations in the 11 lesions were as follows: stop codon mutations, *n* = 6; frameshift mutations, *n* = 2; start codon mutations, *n* = 1; and intron mutations, *n* = 2. There were no sites in which staining was observed in the background mucosa.

### Risk factors for TP53 mutations

The samples were divided into two groups based on the presence or absence of *TP53* mutations (Table 2). *TP53* mutations were identified in 47 of 54 lesions and were not identified in seven lesions. There were no differences with regard to age, sex, invasion depth, tumor diameter, or presence/absence of a flushing reaction. Significant differences were found in the Brinkman index (BI) (*p* = 0.049). *TP53* mutations tended to be associated with a heavy alcohol intake (*p* = 0.052). The association of BI or alcohol intake with *TP53* was not enhanced when the result of p53 immunostaining was added (data not shown). We additionally investigated whether *CYP2A6* polymorphism, which affects smoking-produced nicotine metabolism, alters the association between *TP53* mutational status and smoking [14, 15]. However, no evident correlation between *TP53* and CYP2A6 polymorphism was found (Supplementary Table 3).

**Table 2:** Clinical factors in patients with and without *TP53* mutations

	Mutation detected	Mutation undetected	*P*-value
	*n =* 47	*n =* 7	
Age, year, average ± SD	67.6 ± 8.99	70.0 ± 6.14	0.48
Male. *n* (%)	40 (85%)	5 (71%)	0.33
Invasion depth, IN/EP/LPM/MM/SM1	6/14/19/6/2	1/4/0/2/0	1.00
Size mm, average ± SD	24.7 ± 13.5	20.9 ± 19.4	0.75
Brinkman Index (BI>400), *n* (%)	38 (81%)	3 (43%)	0.049^*^
Cumulative alcohol intake, kg, median ± Q	926 ± 110	303 ± 134	0.052
Flushing response, positive/negative	19/28	1/6	0.24
MCV, fl, mean ± SD	93.4 ± 6.1	95.6 ± 4.2	0.27

Abbreviations: LGIN: low-grade intestinal metaplasia; HGIN: high-grade intestinal metaplasia; EP: invasion limited to the epithelium; LPM: invasion limited to lamina propria mucosae; MM: tumor invading into the muscularis mucosa; SM1: invasion limited to the upper third of the submucosa.

^*^Fisher’s exact test.

### Somatic mutations in cases with multiple lesions

In cases with multiple lesions, the relationship of the patterns of somatic mutations among these lesions were investigated by focusing the analysis on *TP53* mutation. As shown in Table 3, the mutational patterns of *TP53* were different in six of the seven cases involving patients with multiple esophageal lesions, the only exception was case 2 (p.V217G).

**Table 3:** *TP53* mutations in cases with multiple lesions

Case	Lesion	Depth	Var Freq (%)	Mutation
Case 1	1	EP	33.0	p.I162F
	2	LPM	64.9	p.T230fs*6
			65.6	p.I232T
	3	LPM	18.0	p.V173fs*59
			21.9	intron
Case 2	1	HGIN	16.3	p.V217G
	2	LPM	26.2	p.V217G
Case 3	1	EP	76.9	p.A76_S90del15
	2	LPM	61.1	p.P190T
Case 4	1	LPM	25.5	del
	2	LPM	25.5	p.R273H
Case 5	1	LPM	14.3	p.H193R
	2	SM1	73.3	p.Q167*
Case 6	1	HGIN	20.2	p.G105fs*18
	2	LPM	67.9	intron
Case 7	1	EP	34.3	p.R273H
			33.7	intron
	2	EP	20.1	p.I195F

Abbreviations: VF: variant frequency.

## DISCUSSION

In this study, we performed cancer-related gene target sequencing using the Ion Proton platform with a laser-microdissection system to analyze early esophageal neoplasia specimens from patients treated by ESD/EMR. We found that *TP53* mutations already exist in most early lesions, including IEN. CNVs were also already frequent in these early lesions, and the amplification of *PIK3CA* was the most frequent of these CNVs. Smoking was associated with *TP53* mutations in these early lesions.

Clinically, the finding that early ESCC and IEN already have somatic aberrations in cancer-related genes characteristically found in advanced ESCCs has an impact on daily practice. Namely, considering that cancers develop after the accumulation of cancer-related gene alterations, the active treatment of IENs as HGIN or LGIN, which had been classified as premalignant, and treatment had not been actively recommended when their malignant potential was unknown might be recommended based on the information about somatic aberrations.

The high frequency of *TP53* mutations and the resultant high p53 protein expression in ESCC (including early ESCC) has been reported since the 1990s [16–18]. Recently, whole genome and exome NGS of advanced ESCC have confirmed that *TP53* is the most frequently mutated gene; however, most NGS studies investigated advanced lesions [4, 6–8, 10]. In this background, the results of the two most recent NGS studies from China showed the frequent existence of somatic aberrations, including *TP53* gene mutations, even in early dysplastic lesions [3, 9]. However, as mentioned previously, most of the IENs were surgically resected *en bloc* with the adjacent advanced ESCC tissues in these prior studies. Considering the notion of “field cancerization”, it is possible that genomic alterations of advanced ESCCs influence the adjacent IENs; thus, IENs without any advanced ESCCs were more appropriate. In this sense, we focused our analysis on early lesions resected by ESD/EMR without adjacent advanced ESCCs and the LCM technique was introduced for the accurate collection of lesion specimens. The *TP53* mutation rate was found to be significantly high, and almost comparable to the mutation frequency reported in advanced ESCC, even in LGIN- and HGIN-stage lesions. This strongly suggests the significance of the *TP53* gene as a driver gene in early carcinogenesis.

*CDKN2A* was the second most frequently mutated gene in this study. The high frequency of *CDKN2A* mutations was also reported in previous studies, including studies in which NGS was performed [3, 5–8]. Since *TP53* and *CDKN2A* gene products both work as central players to protect cells against uncontrolled proliferation by maintaining surveillance against the induction of DNA damage [19], it is plausible that *CDKN2A* mutations enhance the proliferation of esophageal neoplasia.

In addition to hot spot mutations, CNVs were also frequently observed at the earliest stages of LGIN/HGIN, indicating the importance of CNVs in early esophageal carcinogenesis. *PIK3CA* amplification was the most frequent CNV, and was observed in 48% of early lesions, suggesting the significance of the *PIK3CA* gene in early esophageal squamous cell carcinogenesis. Although *PIK3CA* amplification was also reported in other studies, our study—which used the most accurate LCM system—confirmed that the incidence of *PIK3CA* amplification was high [3, 5–8, 20]. However, the role of CNVs in early carcinogenesis in ESCC should be confirmed in further studies.

Genomic alterations other than *TP53*, *CDKN2A* and *PIK3CA* were also found and accumulated with the progression of the disease in our study (Figure 1, Table 1) as reported previously [3, 9]. Although these secondary aberrant genes showed marked differences according to individual neoplasia, they might work as drivers promoting progression in esophageal carcinogenesis, as these hotspot mutations are registered in the COSMIC database as mutations that are frequently observed in various cancers. However, further clinical as well as basic studies are needed to confirm the role of those secondary aberrations.

Immunohistochemical (IHC) staining of p53 is a commercially available method that can be used to detect p53 abnormalities in the clinical setting. IHC staining of p53 has been used as a surrogate for *TP53* alterations, to support the diagnosis of ESCC. However, since the accuracy of IHC staining of p53 as a surrogate for *TP53* alterations has not been investigated in the era of NGS, we compared samples that were IHC-positive for p53 to samples in which *TP53* alterations were detected by NGS. In a previous study of 64 locally-advanced ESCCs, the detectability of *TP53* aberrations by PCR-SSCP-based mutation analysis and IHC was reported to be 31% and 66%, respectively; thus, IHC was superior for detecting *TP53* aberrations [21]. However—as demonstrated in our study—the NGS-based mutational analysis applied in the present study was superior to IHC for detecting *TP53* aberrations. When we investigated the *TP53* mutation profiles in the 11 neoplasia samples that were mutation-positive but IHC-negative, most samples had genetic aberrations (stop codon mutations, frameshifts, start codon and intron mutations) that could abrogate the expression of p53 protein. Thus, it is plausible that the p53 protein was not detected by IHC. On the other hand, in five neoplasia samples, *TP53* aberrations were only detected by IHC. We suspect that this was because the *TP53* mutations were not covered by this specific panel. Based on these results, we propose that the combination of these two tests might be more accurate for detecting *TP53* aberrations in the clinical setting.

In addition to neoplasia lesions, we also evaluated the adjacent background mucosa to determine the variant status. With the exception of one specimen (ESCC_42) no evident aberrations (above the cut-off level) were detected (Supplementary Table 1). On the other hand, the possible existence of genomic aberrations in the background tissues at low frequencies cannot be denied, as some variants were repeatedly observed across different background mucosa specimens (Pro72Ala of TP53, and Val578del of Notch1); further studies are needed will be needed to confirm this.

With regard to the cause of cancer-related gene aberrations, we focused our analysis on *TP53* mutations. As shown in Table 2, smoking was extracted as risk factors for *TP53* mutations in early esophageal neoplasia, which is compatible with previous studies. Although the SNP of *CYP2A6*, a gene responsible for nicotine metabolism [14, 15], had no evident influence on *TP53* aberrations, a study targeting larger number of patients is still needed to clarify the role of *CYP2A6* and nicotine in esophageal carcinogenesis (Supplementary Table 3) [22]. Nucleotide substitution analysis revealed the high frequency of C>A transversion, which is known to be highly associated with benzo(a)pyrene-induced carcinogenesis [23]. However, the most frequent mutational pattern was G>A transition, which is considered to occur due to the enzymatic activity of *APOBEC* family genes [3, 4, 6–8], which are generally induced by chronic inflammation. Indeed, in a recent NGS study, G>A transition was frequently observed in early lesions, confirming this result. On the other hand, the role of chronic inflammation and/or the *APOBEC* family proteins in ESCC carcinogenesis and their potential correlation with smoking, drinking and other causes, such as human papilloma virus infection, are not well understood, and further studies are needed to confirm the fundamental molecular mechanisms that induce *TP53* mutations.

The use of a commercially-available compact panel that only targets 50 cancer-related genes was one limitation of the present study. On the other hand, the frequent presence of hotspot mutations and CNVs in cancer-related genes were successfully reproduced using the panel in the early esophageal neoplasm specimens in this study. Another limitation of the present study is that the results determined by the Ion Reporter algorithm in the analysis of CNV were not confirmed further by other methods. However, recent studies on CNVs reported that the CNVs detected by CNV cancer panels were highly consistent with the CNVs detected by other methods (*e.g.,* Tiling array, or FISH), suggesting the high reliability of this algorithm.

In conclusion, using early esophageal squamous neoplasia accurately-collected with LCM from endoscopically- resected tissues, we could successfully perform Ion Proton-based sequencing of targeted cancer-related genes. We revealed the high frequency of *TP53*/*CDKN2A* hotspot mutations and *PIK3CA* copy number amplification, which was comparable to the frequency observed in advanced ESCC. These findings might provide a molecular basis for the treatment of these early lesions.

## MATERIALS AND METHODS

### Patients and samples

**Figure 5:**
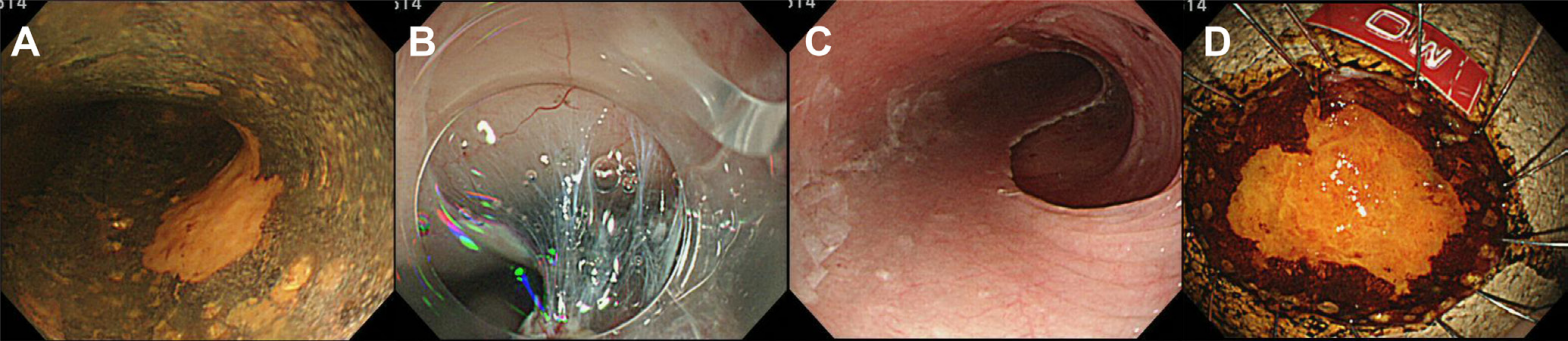
Endoscopic submucosal dissection (ESD) of esophageal neoplasia: a representative procedure (**A**) Early esophageal neoplasia was diagnosed by iodine staining as an “unstained area”. (**B**) The submucosal layer seen during mucosal dissection. (**C**) The esophageal lumen immediately after ESD. (**D**) The resected specimen.

Fifty-four esophageal squamous cell neoplastic lesions from 47 consecutive cases in which resection was performed by ESD or EMR at Yamanashi University Hospital from 2012 to 2014 were included in the study. Their background clinical information, which was collected retrospectively from medical records, is shown in Table 4. Initially, 55 lesions were included; however, one lesion was excluded because of its inadequate FFPE quality. Endoscopic resection was conducted based on the preoperative diagnosis; an example of ESD is shown in Figure 5. The histological diagnoses of these 54 lesions determined after ESD/EMR, according to the *Japanese Classification of Esophageal Cancer (10th edition)* [24], were as follows: low-grade intraepithelial neoplasia (LGIN, *n* = 1); high-grade intraepithelial neoplasia (HGIN, *n* = 6); invasion limited to intraepithelial carcinoma (EP, *n* = 18); invasion limited to the lamina propria mucosae (LPM, *n* = 19); invasion limited to the muscularis mucosae (MM, *n* = 8); and invasion limited to the submucosal layer within 200 μm from the muscularis muscle plate (SM1, *n* = 2) (Table 4). If neoplasia was found to have infiltrated into the MM or deeper after the histological examination of resected specimen, we conducted additional resection to achieve complete resection. As shown in Table 4, additional surgical resection was performed for 8 MM lesions and 2 SM1 lesions for which the infiltration had been misdiagnosed as no deeper than LPM before ESD/EMR. Computed tomography, endoscopic ultrasonography and the histological examination of resected specimens were performed to confirm that all cases were negative for N and M factors. Thus, there were seven patients with premalignant lesions (LGIN and HGIN), 45 patients with stage 0 lesions (EP, LPM, and MM), and two patients with Stage I lesions (SM1) in this study.

**Table 4:** Characteristics of the 54 esophageal neoplasia lesions

	*n =* 54
Age, year, average ± SD	67.9 ± 8.66
Male (%)	45 (83%)
Invasion depth, LGIN/HGIN/EP/LPM/MM/SM1	1/6/18/19/8/2
Size, mm, average ± SD	24.2 ± 14.2
Smoking, None/smoker/heavy smoker	9/36/9
Alcohol, None/light drinker/heavy drinker^*^	4/16/34
Flushing response, Positive/negative/NA	20/23/11
MCV, fl, mean ± SD	93.8 ± 5.87

Abbreviations: LGIN: low-grade intraepithelial neoplasia; HGIN: high-grade intraepithelial neoplasia; EP: invasion limited to the epithelium; LPM: invasion limited to lamina propria mucosae; MM: tumor invading into the muscularis mucosa; SM1: invasion limited to the submucosal layer within 200 μm from the muscularis mucosa.

^*^Heavy drinker is defined as an ethanol intake of ≥ 40 g/day.

The study protocol conformed to the ethical guidelines of the 2000 Declaration of Helsinki and the patients gave their informed consent for participation in the study, which was approved by the Human Ethics Review Committee of University of Yamanashi.

### Laser capture microdissection and DNA extraction

FFPE blocks were continuously cut into 3-µm-thick sections for the detection of tumor and background non-tumor tissues, and into 8-µm-thick sections for laser-microdissection. The sections were then stained with hematoxylin and eosin (HE). Tumor tissue (T) and non-tumor tissue (N) were accurately laser-microdissected from 8-µm-thick section slides with reference to 3-µm-thick HE-stained section slides using an ArcturusXT^™^ Laser Capture Microdissection System (Thermo Fisher, Waltham, MA, USA). DNA from the resected tissues was extracted using a QIAamp DNA FFPE kit (QIAGEN, Milan, Italy) according to the manufacturer’s instructions. The quantity and quality of the extracted DNA were assessed using a NanoDrop (Thermo Fisher).

### Multiplex PCR of targeted genes and deep sequencing

We used a ready-made gene panel (Ion AmpliSeq Cancer Hotspot Panel v.2, Thermo Fisher) to amplify 50 cancer-related target genes. The panel contained 207 primer pairs and targets 2,790 hotspot mutations in the following 50 cancer-related genes in the COSMIC database 8: *ABL1, AKT1, ALK, APC, ATM, BRAF, CDH1, CDKN2A, CSF1R, CTNNB1, EGFR, ERBB2, ERBB4, EZH2, FBXW7, FGFR1, FGFR2, FGFR3, FLT3, GNA11, GNAS, GNAQ, HNF1A, HRAS, IDH1, JAK2, JAK3, IDH2, KDR/VEGFR2, KIT, KRAS, MET, MLH1, MPL, NOTCH1, NPM1, NRAS, PDGFRA, PIK3CA, PTEN, PTPN11, RB1, RET, SMAD4, SMARCB1, SMO, SRC, STK11, TP53,* and *VHL*.

Briefly, 20 ng of DNA was amplified by a polymerase chain reaction (PCR) using the abovementioned primer panel and AmpliSeq^™^ HiFi Master Mix (Ion AmpliSeq^™^ Library Kit, Thermo Fisher). The multiplexed amplicons were treated with FuPa Reagent (Thermo Fisher) for partial digestion of the primer sequences and phosphorylation. The amplicons were then ligated to adapters from an Ion Xpress^™^ Barcode Adapters 1–96 Kit (Thermo Fisher), according to the manufacturer’s instructions. After ligation, the amplicons underwent nick-translation and additional library amplification by a PCR, in order to complete the linkage between the adapters and amplicons. The size and concentration of the amplicon libraries that were produced were then checked using a High Sensitivity DNA Kit (Agilent, Santa Clara, CA, USA) on an Agilent 2100 Bioanalyser on-chip electrophoresis system.

Multiplexed barcoded libraries were amplified by an emulsion PCR on Ion Sphere^™^ particles (ISPs) and sequencing was performed on an Ion Chef^™^ System and an Ion Proton^™^ Sequencer (Thermo Fisher) using the Ion PI Hi-Q Chef Kit (Thermo Fisher) according to the manufacturer’s instructions. The Torrent Suite Software v.4.0 (Thermo Fisher) was used to align reads to the hg19 reference genome and to generate run metrics, including total read counts and quality. Variant Caller v.4.0 (Thermo Fisher) was used to identify variants.

A total of 2,790 hotspot mutations of the 50 cancer-related genes were reported in the Catalogue of Somatic Mutations in Cancer (COSMIC; hotspot mutations) database 8. The cut-off variant frequency per read depth was set at 5% [25]. Variants were regarded as neoplasia-specific if they were only detected in neoplasia (not in the adjacent background mucosa) and if the variant frequency was over the cut-off value of 5%.

Copy number variations (CNVs) were detected using Ion Reporter^™^ Software Copy Number Variation Analysis (Thermo Fisher). The CNV detection algorithm was based on a hidden Markov model, and CNVs with confidence scores of ≥ 10 were included in the analysis, as reported in a previous study [26].

### Immunohistochemistry

Immunostaining of 2-μm thin-sections of specimens that were continuous with the DNA-extracted sections was performed. The sections were deparaffinized, immersed in an antigen activation solution (Target Retrieval Solution, High pH), and autoclaved at 120°C for 20 minutes. P53-specific antibody (Anti-Human p53 Protein, Clone DO-7; DAKO) was used as the primary antibody and Envision^™^ plus Dual Link HRP (DAKO) was used as the secondary antibody. After color development with Diaminobenzidine, counterstaining with hematoxylin was performed and the staining properties were assessed. S.K, the first author of this manuscript, blindly assessed the p53 immunostaining results, without information about *TP53* aberrations, with the advice of two pathologists.

### Statistical analysis

The Mann-Whitney *U* test or Fisher’s exact test was used to compare demographic variables between two groups. The Cochran-Armitage trend test was used to detect trends in multiple groups. Odds ratios and 95% confidence intervals were calculated. Two-tailed *P* values of < 0.05 were considered to indicate statistical significance.

## SUPPLEMENTARY MATERIALS FIGURE AND TABLES


