# Supplementary material for: Target sequencing of cancer-related genes in early esophageal squamous neoplasia resected by endoscopic resection in Japanese patients
Source: Oncotarget. 2018 Dec 4;9(95):36793–803. doi: 10.18632/oncotarget.26397 (PMC6298401; doi:10.18632/oncotarget.26397)
Supplement: Supplementary file 1 [file oncotarget-09-36793-s001.pdf]

Target sequencing of cancer-related genes in early esophageal squamous neoplasia resected by endoscopic resection in Japanese patients

SUPPLEMENTARY MATERIALS

Supplementary Table 1: Mutations detected in adjacent normal tissues

				Tumor tissue		Adjacent normal tissue	
Cases	Depth	Gene	Mutation	Coverage	VF (%)	Coverage	VF (%)
ESCC_10	EP	*TP53*	p.Phe270Leu	1638	2.1	2000	3.1
ESCC_16	EP	*TP53*	p.Arg273Cys	1997	45.9	141	0.7
ESCC_17	EP	*APC*	p.Gln1294Ter	316	5.4	79	1.3
ESCC_26	EP	*TP53*	p.Pro72Ala	1999	2.8	2000	1.4
ESCC_3	EP	*NOTCH1*	p.Val1578del	1947	3.0	1941	1.2
ESCC_3	EP	*PTEN*	p.Pro246Leu	1994	7.1	1990	1.9
ESCC_36	EP	*TP53*	p.Pro72Ala	711	3.7	278	2.9
ESCC_4	EP	*TP53*	p.Arg342Ter	1999	2.7	1999	1.5
ESCC_4	EP	*EGFR*	p.Gly721Ser	1999	4.2	2000	1.0
ESCC_40	EP	*TP53*	p.Arg110Leu	1994	21.3	1993	1.3
ESCC_1	LPM	*NOTCH1*	p.Val1578del	1931	1.6	1137	0.1
ESCC_18	LPM	*TP53*	p.Pro72Ala	1999	2.9	228	3.1
ESCC_21	LPM	*NOTCH1*	p.Val1578del	1958	2.7	1040	1.2
ESCC_21	LPM	*TP53*	p.Glu198Ter	1995	53.7	1410	0.1
ESCC_24	LPM	*TP53*	p.Arg110Cys	740	2.2	1999	1.3
ESCC_32	LPM	*TP53*	p.Pro278Ser	2000	72.8	2000	1.6
ESCC_34	LPM	*TP53*	p.Pro72Ala	1999	3.0	1999	3.7
ESCC_38	LPM	*TP53*	p.Pro72Ala	1997	4.3	1841	3.9
ESCC_5	LPM	*RB1*	p.Arg556Gln	1999	3.7	94	1.1
ESCC_20	MM	*SMAD4*	p.Glu337Gly	502	3.2	354	1.1
ESCC_41	MM	*TP53*	p.Arg306Ter	2000	38.9	1356	1.2
ESCC_42	MM	*TP53*	p.Pro72Ala	1999	4.5	1991	5.9
ESCC_52	MM	*TP53*	p.Pro72Ala	2000	4.6	2000	4.3
ESCC_19	SM1	*TP53*	p.Arg213Ter	1963	2.9	1978	1.0
ESCC_19	SM1	*CDKN2A*	p.Arg80Ter	1997	2.4	1995	1.3

Abbreviations: VF: variant frequency; EP: invasion limited to the epithelium; LPM: invasion limited to lamina propria mucosae; MM: tumor invading into the muscularis mucosa; SM1: invasion limited to the upper third of the submucosa.

Supplementary Table 2: Gene alterations between IEN and superficial ESCC

	IEN	EP/LPM/MM	*P*-value	
	(*n* = 7)	(*n* = 47)		
Mutation				
TP53	6	39	0.60	a
CDKN2A	2	9	0.98	a
TP53/CDKN2A	6	41	0.81	a
CNV				
Average number of CNV	2.571	3.822	0.47	b

Abbreviations: a, Yates's correction for continuity; b, *t*-test.

Supplementary Table 3: Association between *CYP2A6* polymorphisms and *TP53* mutation

	Mutation detected	Mutation undetected	*P*-value
	(*n* = 47)	(*n* = 7)	
Brinkman Index (BI), median ± Q	38 (81%)	3 (43%)	0.049*
CYP2A6, GG/GA+AA/undetermined	31/9/7	5/2/0	0.726

*Fisher's exact test

In CYP2A6 SNP analysis, TaqMan SNP assay (SNP ID: rs1137115, Thermofisher) was performed.

	Early lesons<br>This study (n=54)<br>Mutation (%)	Advanced ESCCs<br>Database (n=227)<br>Mutation (%)	
TP53	87.0	68.7	**
CDKN2A	20.4	3.5	**
APC	5.6	1.8	
PTEN	3.7	3.5	
KDR	3.7	2.2	
STK11	3.7	0.9	
KIT	3.7	0.9	
CSF1R	3.7	0.9	
VHL	3.7	0	*
RB1	1.9	7.9	
PIK3CA	1.9	6.6	
SMARCB1	1.9	0.9	
BRAF	1.9	0.9	
FLT3	1.9	0.4	
EGFR	1.9	0.4	
CTNNB1	1.9	0.4	
PDGFRA	1.9	0	
HRAS	1.9	0	
FGFR3	1.9	0	
NOTCH1	0	8.4	
FBXW7	0	3.5	
ERBB4	0	2.6	
SMO	0	1.8	
PTPN11	0	1.3	
ATM	0	1.3	
ALK	0	1.3	
MLH1	0	0.9	
JAK2	0	0.9	
FGFR2	0	0.9	
CDH1	0	0.9	
NPM1	0	0.4	
MET	0	0.4	
HNF1A	0	0.4	
FGFR1	0	0.4	
EZH2	0	0.4	
ABL1	0	0.4	
SRC	0	0	
SMAD4	0	0	
RET	0	0	
NRAS	0	0	
MPL	0	0	
KRAS	0	0	
JAK3	0	0	
IDH2	0	0	
IDH1	0	0	
GNAS	0	0	
GNAQ	0	0	
GNA11	0	0	
ERBB2	0	0	
AKT1	0	0	

* Fisher's exact test

Supplementary Figure 1: The somatic aberration status between our early esophageal neoplastic lesions and advanced ESCCs (internet-based data) was compared. The somatic aberration data of 227 advanced ESCCs in 50 cancer-related genes analyzed in our study was retrieved from the online database (cBioPortal for Cancer Genomics [http://cbioportal.org]: mutation data of the Cancer Genome Atlas [TCGA] and International Cancer Genome Consortium [ICGC] were available) [13]. The only evident difference between these two groups was in the expression of three genes: *CDKN2A*, *TP53*, and *VHL*.
